# Thailand – how far are we from achieving a healthy and sustainable diet? A longitudinal ecological study

**DOI:** 10.1016/j.lansea.2024.100478

**Published:** 2024-09-13

**Authors:** Alice Beckmann, Carola Strassner, Karunee Kwanbunjan

**Affiliations:** a Department of Food Nutrition Facilities, FH Münster University of Applied Sciences, Corrensstraße 25, Münster, 48149, Germany; b Department of Tropical Nutrition and Food Science, Faculty of Tropical Medicine, Mahidol University, 10400, Bangkok, Thailand

**Keywords:** Planetary health diet, Sustainable diets, Nutrition transition, Thailand's food systems, Food consumption, Dietary recommendations for Thailand, Thaifood

## Abstract

**Background:**

Newly industrialized countries like Thailand have been influenced by globalization, westernization, and urbanization over the last decades, leading to changes in dietary habits as well as food production. Consequences of these changes include rising non-communicable diseases (NCDs) and environmental degradation, which are defined as the leading global challenges today. The objectives of this study are to identify Thailand's dietary changes, considering health and sustainability aspects, and to determine correlations between these changes and NCD cases as well as environmental impacts (GHG emissions, land-, nitrogen-, phosphorus-use). In this way, diet-related adjustments can be identified to promote planetary and human health.

**Methods:**

In this longitudinal ecological study, relative differences between the average food consumption in Thailand and the reference values of a healthy and sustainable diet, the Planetary Health Diet (PHD), were calculated. Furthermore, a bivariate correlation analysis was conducted, using data, based on Food and Agriculture Organization's (FAO's) data, results from the Global Burden of Disease Study (GBD), and PHD's reference values.

**Findings:**

The consumption quantities of meat, eggs, saturated oils, and sugar increased significantly since 1961. The food groups, that have exceeded PHD's upper reference values, include sugar (+452%), red meat (+220%), grains (+143%), saturated oils (+20%) and eggs (+19%), while vegetables (–63%), and unsaturated oils (–61%) have fallen below PHD’s lower limits. Concerning the bivariate correlation analyses, all investigated variables show significant correlations. The most significant correlations were found in NCD cases (r = 0.903, 95% CI 0.804–0.953), nitrogen use (r = 0.872, 95% CI 0.794–0.922), and land use (r = 0.870, 95% CI 0.791–0.921), followed by phosphorus use (r = 0.832, 95% CI 0.733–0.897), and green-house gas (GHG) emissions (r = 0.479, 95% CI 0.15–0.712).

**Interpretation:**

The results show, that the determined differences of unhealthy or unsustainable food groups have increased concurrently with NCD cases and environmental impacts over the last decades in Thailand. A shift towards a reduced intake of sugar, red meat, grains, saturated oils and eggs along with an increase in vegetables and unsaturated oils, might support environmental and human health.

**Funding:**

None.

**Research in context**

**Evidence before this study**

Numerous studies have shown that global dietary habits have changed, especially in countries with rising Gross Domestic Product (GDP). These changes include higher consumption of highly processed foods, including animal products like meat, salt, sugar, and saturated oils. It is also well known that cases of NCDs and environmental changes have increased worldwide. Thereby, dietary changes are linked to rising NCDs and environmental degradation. To illustrate to what extent the world diverges from a healthy and sustainable diet, a current study shows percentage differences between the Planetary Health Diet (PHD) and average consumption levels across all continents. However, this study does not account for changes over recent decades nor do they present country-specific analyses. A study by Liu et al. shows, that economic growth is associated with a decreasing intake of quality nutrients (proteins, vitamins, minerals) and an increasing intake of disqualified nutrients (total fats, saturated fats, and added sugars) in several countries, thus promoting diet-related diseases as well as environmental impacts, such as climate change and biodiversity loss.

**Added value of this study**

Building on this knowledge, the present study focuses on Thailand, a country with rising GDP, and specifically includes dietary changes over the last decades. Thereby, percentage differences between the average diet in Thailand and the PHD's reference values has been calculated over the last 60 years. This allows for a comprehensive picture of the average diet in Thailand and its changes over time, including health and environmental aspects. Furthermore, this study incorporates various aspects, including changes in dietary habits, health status (NCDs), as well as environmental impacts (GHG emissions, land use, nitrogen, phosphorus) over the last decades, and connects them. The calculated correlations show the extent to which deviations from a sustainable and healthy diet (PHD) are associated with environmental changes and NCD cases over time. Thereby, the use of percentage differences, between dietary pattern and PHD's reference values, as an independent variable is novel. This study is also unique in that it links these dietary-, health- and environmental-aspects over several decades in Thailand, a country with rising GDP. Since Thailand's dietary habits, and their deviations from the PHD are analyzed across 14 food groups, a comprehensive and detailed overview can be presented. In this way, dietary interventions can be identified that might support human and planetary health.

Our results show, that the intake of unhealthy and unsustainable food groups increased over the last decades in Thailand. Especially the intake of sugar, red meat, grains, saturated and unsaturated oils as well as vegetables diverge from PHD's reference values. Furthermore, these dietary changes, correlate with environmental impacts and NCD cases in Thailand. This study thus supports results from previous studies indicating that dietary changes are linked to rising NCD cases and environmental degradation. Our results also support findings from previous studies linking negative dietary changes with an increase in GDP.

**Implications of all the available evidence**

Our findings can support the formulation of current dietary guidelines in Thailand, taking human and planetary health into account. These may include a reduction in sugar, red meat, grains, saturated oils and eggs, along with an increase in vegetables, and unsaturated oils. The unexpectedly high consumption of sugar highlights the importance of reducing the intake significantly. Policy makers, in the fields of public health and agriculture, can benefit from the results, especially in Thailand, South East Asia or countries with rising economic growth. Consequently, the findings can contribute to the development of new agricultural and public health strategies, as well as current dietary guidelines. For more precise results, that consider the origin and preparation of food groups and do not rely on supply data, further research could be conducted, including consumption surveys. In addition, advanced studies could focus on further countries or planetary boundaries, particularly regarding biodiversity loss. In this case, our method, presented in the current study, could be applied for future research.

## Introduction

### Status Quo world and Asia

The development towards increasing numbers in NCDs, GHG emissions, land use changes, and other negative impacts on human^1^, ^2^, ^3^, ^4^, ^5^, ^6^, ^7^, ^8^ and planetary health has globally increased over the last decades, especially due to globalization and urbanization.^2^, ^3^, ^4^, ^5^^,^^7^, ^8^, ^9^ Thereby, food production and dietary habits are the main driver for global environmental changes and health issues.^7^ Globally, 20–25% of all death among adults is linked to dietary imbalances.^10^ Concurrently, agriculture occupies around 40% of the terrestrial surface^11^ and is responsible for a third of global GHG emissions.^12^ Furthermore, the overapplication of phosphorus and nitrogen in global agriculture is causing eutrophication, a factor promoting biodiversity loss and climate change.^13^, ^14^, ^15^ A reduction in animal-based foods can thereby significantly contribute to reducing premature mortality, GHG emissions, cropland use as well as nitrogen and phosphorus application.^7^^,^^16^ The effects are particularly evident in high-income and upper-middle-income countries.^16^ Because of the rapid economic growth in Asia, the transition from diets, dominated by low fat and high fiber foods, to increased consumption of animal-based, processed, packaged, and convenience foods is taking place particularly fast.^1^^,^^17^^,^^18^ This transition has led to a higher intake of saturated oils and Trans-fatty acids, salt as well as sugar.^17^^,^^18^ Foods which are associated with the development of NCDs.^1^^,^^6^ Furthermore, the meat consumption in Asia increased constantly over the last decades.^17^, ^18^, ^19^, ^20^ In 2029, Asia is expected to account for around 56% of global meat trade,^21^ which might lead to further rising NCD cases as well as environmental degradation.^10^^,^^22^ Especially upper- and middle-income countries in Asia with rising economic growth are affected by these changes.^1^^,^^6^ A current study by Liu et al. shows, that economic growth is associated with a decreasing intake of quality nutrients (proteins, vitamins, minerals) and an increasing intake of disqualified nutrients (total fats, saturated fats, and added sugars) in several countries, thus promoting diet-related diseases as well as environmental impacts.^6^

### Status Quo Thailand

Thailand has experienced significant economic growth over the last decades and is considered as one of the upper- and middle-income countries in Asia.^1^ In 2022, Thailand reached a Gross Domestic Product (GDP) of 495 billion US$, compared to 3 billion US$ in 1961.^23^ The rapid economic growth has led to wealth and decreasing deaths from communicable diseases or maternal, prenatal and nutrition conditions.^24^^,^^25^ Nevertheless, Thailand's adult mortality has not fallen sharply over the last decades. While the risks of household air pollution, child growth failure, unsafe water and sanitation decreased, high systolic blood pressure, high fasting plasma glucose, high body-mass index (BMI), are now considered the leading health risk factors.^24^^,^^25^ As a result, numbers of NCDs in Thailand are increasing and accounted for 74% of total mortality in 2016, with leading causes of cardiovascular diseases (23%), cancers (18%), chronic respiratory diseases (6%) and diabetes (4%).^26^ This development is associated with shifting dietary habits with an increasing intake of animal products and processed foods, high in saturated fats, salt and sugar.^17^^,^^18^^,^^27^, ^28^, ^29^, ^30^, ^31^, ^32^ Reasons for these changes are rising western-style fast food restaurants and conventional supermarkets, which has gained popularity among Thai people, as a result of intensive marketing campaigns.^27^ Additionally, more than half of the population in Thailand is living in urban areas today. The rising urbanization is associated with higher numbers of NCDs, since rural Thai people tend to live closer to nature with higher physical activity levels, fresh water and lower consumption of processed foods, sugar, starch and fat.^33^^,^^34^ Conversely, traditional Thai cuisine includes a wide variety of diverse, fresh foods such as whole grain rice, fish, seafood, tropical fruits, coconuts, as well as health promoting herbs and spices.^17^^,^^34^, ^35^, ^36^ Due to a less industrialized food production in the past, fewer highly processed foods were consumed, resulting in lower intake of sugar and saturated oils as well as higher intake of fiber.^35^

Beside of dietary changes and simplified access to highly processed fast food, Thailand's agriculture changed over the last decades. As a result, land-use changes and GHG emissions increased as well as the use of pesticides, nitrogen and phosphorus.^2^, ^3^, ^4^^,^^37^ These changes promote soil degradation, eutrophication, climate change as well as biodiversity loss, which makes them a significant threat to the environment.^8^^,^^38^^,^^39^

### Objectives

The present work intends to examine Thailand's dietary changes between 1961 and 2020, related to NCD cases and environmental changes, concerning four planetary boundaries that have already been exceeded (GHG emissions, land use changes, nitrogen- and phosphorus use).^7^ Thereby, it should be analyzed whether correlations exist between dietary changes and NCD cases, as well as four environmental impacts. To assess in which way Thailand's average food intake in the past and present differs from the guidelines of a sustainable and healthy diet, these two variables are compared by calculating percentage differences. In this case, the guidelines of EAT-Lancet Commission's PHD are used. The diet's goal is to feed 10 billion people in 2050 without threatening human and environmental health. More specifically, the PHD aims to significantly contribute to staying within the planetary boundaries and reducing diet-related diseases (caused by both overnutrition and undernutrition) and is therefore used as a definition for a sustainable and healthy diet in this study. The diet is based on 14 macronutrient reference values with an average amount of 2500 kcal per day and capita, but can vary due to ranges (see Table 1). Since plant-based foods are associated with less environmental and health outcomes, especially the intake of whole grains, fruits, vegetables and unsaturated oils are recommended, while animal-based foods should be consumed in smaller amounts (see Table 1). The specific consumption quantities, along with a focus on sustainability aspects, give the PHD an advantage over other well-known diets for this study, such as the Mediterranean diet.^7^

**Table 1:** Thai Food Pattern (TFP) in 1961 and 2020, compared to PHD's reference diet (g/capita/day).

	TFP 1961	TFP 2020	PHD
**Whole grains**			
Rice, wheat, corn and other	393	565	232
**Tubers or starchy vegetables**			
Potatoes and cassava	10	29	50 (0–100)
**Vegetables**			
All vegetables	114	111	300 (200–600)
**Fruits**			
All fruits	188	177	200 (100–300)
**Dairy Foods**			
Whole milk or equivalents	7	39	250 (0–500)
**Protein sources**			
Beef, lamb and pork	35	45	14 (0–28)
Chicken and other poultry	9	31	29 (0–58)
Eggs	25	33	13 (0–25)
Fish	24	80	28 (0–100)
Legumes	2	13	75 (0–100)
Nuts	83	27	50 (0–75)
**Added fats**			
Unsaturated oils	2	16	40 (20–80)
Saturated oils	2.4	14.2	11.8 (0–11.8)
**Added sugars**			
All sugars	11	171	31 (0–31)

Previous analysis, has already shown, that the average dietary pattern in Asia differs strongly from the maximum recommendations of the PHD in 2018, especially in red meat (252%) as well as red and processed meat (327%). At the same time, the average intake of plant-based foods in Asia shows strong differences in regard to the minimum recommendations of the PHD with −45% in fruits and vegetables, −71% in nuts, and −74% in legumes.^40^ Another study, based on the Global Dietary Database, presents the average consumption quantities of animal-based foods worldwide between 1990 and 2018. However, this research does not include references to the PHD or environmental aspects.^41^ To generate country-specific and current results for Thailand, percentage differences within the framework of PHD's 14 food groups were calculated in this study. They are providing a comprehensive insight into Thailand's dietary pattern, taking environmental and health aspects under account. By identifying specific needs for change, based on the examined food groups, sustainable development can be supported. Furthermore, it should be determined if Thailand's dietary changes correlate with NCD cases and environmental impacts. A comparison between the average food intake in Thailand, based on FAO's supply data, and PHD's reference values, over the last decades has not been undertaken. Furthermore, connections between dietary changes and NCD cases as well as environmental changes in Thailand has not been implemented yet. The present study aims to address this research gap by identifying potential dietary pattern that are leading to negative environmental and health effects. Accordingly, the research question is formulated as follows:

In which way have the average consumption quantities and dietary habits of 14 food groups changed between 1961 and 2020 in Thailand, do these changes correlate with Thailand's NCD cases and environmental changes regarding four planetary boundaries (GHG emissions, land use changes, nitrogen- and phosphorus use) and to what extent do the consumption quantities differ from the recommendations of the PHD?

The results can support policy makers, especially those who are working on current dietary guidelines and public health concepts in Thailand or South East Asia.

## Methods

### Study design

To identify in which way Thailand's dietary habits have changed over the last 60 years and to find explanations for these shifts, a qualitative literature review was conducted. This review is specific to the current study and has not been previously published. Furthermore, this longitudinal ecological study is using quantitative methods to identify dietary changes between 1961 and 2020. The changes were linked to the concept of a healthy and sustainable diet, by calculating percentage differences between Thailand's dietary pattern and PHD's reference values (mid-points). Additionally, correlations between the previously calculated differences of unhealthy or unsustainable food groups and NCD cases as well as environmental impacts were calculated. In this way it can be determined, if the respective variables followed a similar trend over time.

### Data collection

The study is mainly based on data provided by FAO. An important reason for selecting FAO's data, is the systematically, standardized, and country specific data collection since 1961. Furthermore, FAO's data allow a comprehensive overview of Thailand's dietary pattern (based on supply data), since the whole population is considered, including all age groups, genders, ethnic origins and social classes. The datasets can illustrate shifts in dietary pattern and agricultural practices, both aspects, which are analyzed and compared in this study. Since economic growth and modernization in Thailand, particularly started during the 1960s, significant changes in food habits and agricultural reforms began from this period onwards.^2^, ^3^, ^4^^,^^17^^,^^18^^,^^42^ Therefore, FAO's data and the corresponding timeframe are suitable for this study. Concerning the health status in Thailand, results from the “Global Burden of Disease Study (GBD)", provided by the “Institute for Health Metrics and Evaluation (IHME)", were used, which describe DALYs due to NCD cases.^43^ IHME and FAO provide data for both genders and all age groups, enabling a comparison between the calculated percentage differences and DALYs due to NCDs.

### Percentage differences

To capture the evolution of dietary pattern in Thailand, initially, FAO's supply data of different food items were summed together for each year between 1961 and 2020, to illustrate them in PHD's 14 food groups. Subsequently, the results are converted to g/capita/day, making a comparison between FAO's supply data and PHD's reference values possible.

To identify to which extent these previously calculated dietary changes diverge from EAT-Lancet Commission's reference values (PHD's mid-points), percentage differences between these two variables were calculated for each year between 1961 and 2020. The mathematical formula is expressed as follows:^44^

Percentage Difference (%) = ((FAO's food supply (g/capita/day)—PHD mid-point (g/capita/day))/PHD mid-point (g/capita/day)) x 100%

**Fig. 1:**
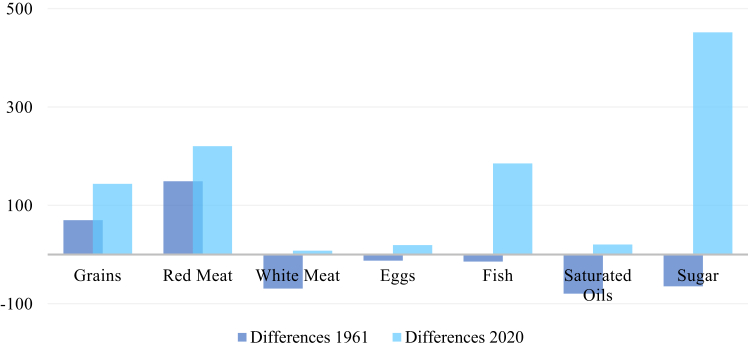
**Positive differences between PHD's mid-points and Thai foodpatternin %**. (Source: own source, based on EAT-Lancet-Commissions and FAOs data).

**Fig. 2:**
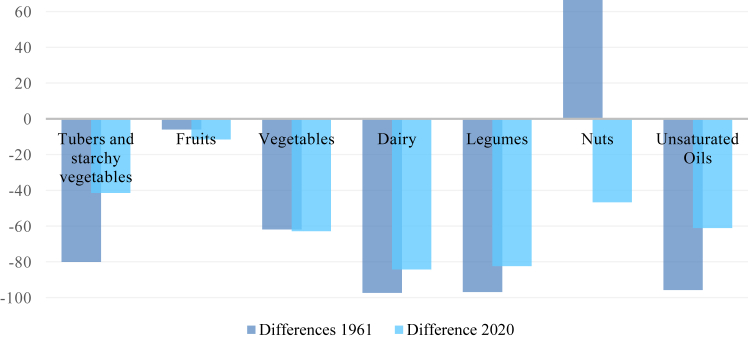
**Negative differences between PHD's mid-points and Thai foodpatternin %**. (Source: own source, based on EAT-Lancet-Commissions and FAOs data).

Figs. 1 and 2 illustrate the positive and negative differences of each food group in 1961 and 2020. Thereby, comparisons between the respective food groups and two different stages (beginning of economic growth and current stage) can be assessed.

### Correlations

**Fig. 3:**
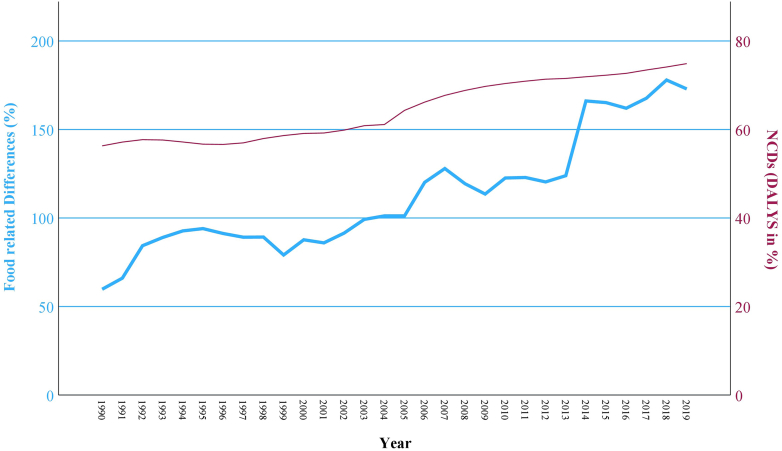
**Development NCDs and food related differences**. (Source: own source, based on EAT-Lancet-Commissions, FAOs and IHMEs data).

**Fig. 4:**
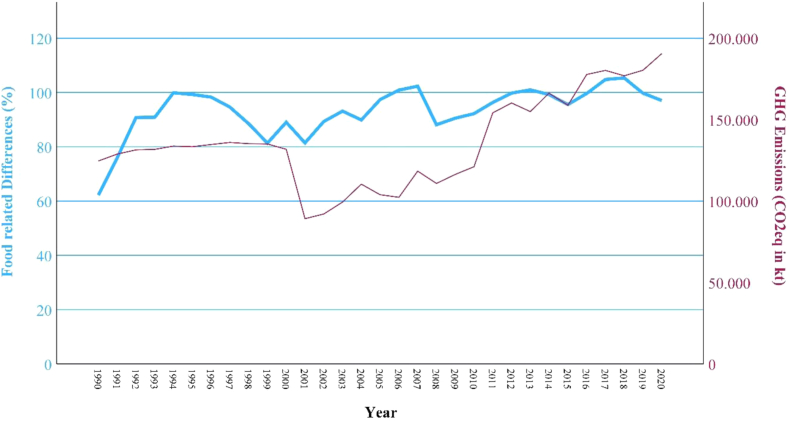
**Development GHG emissions and food related differences**. (Source: own source, based on EAT-Lancet-Commissions and FAOs data).

**Fig. 5:**
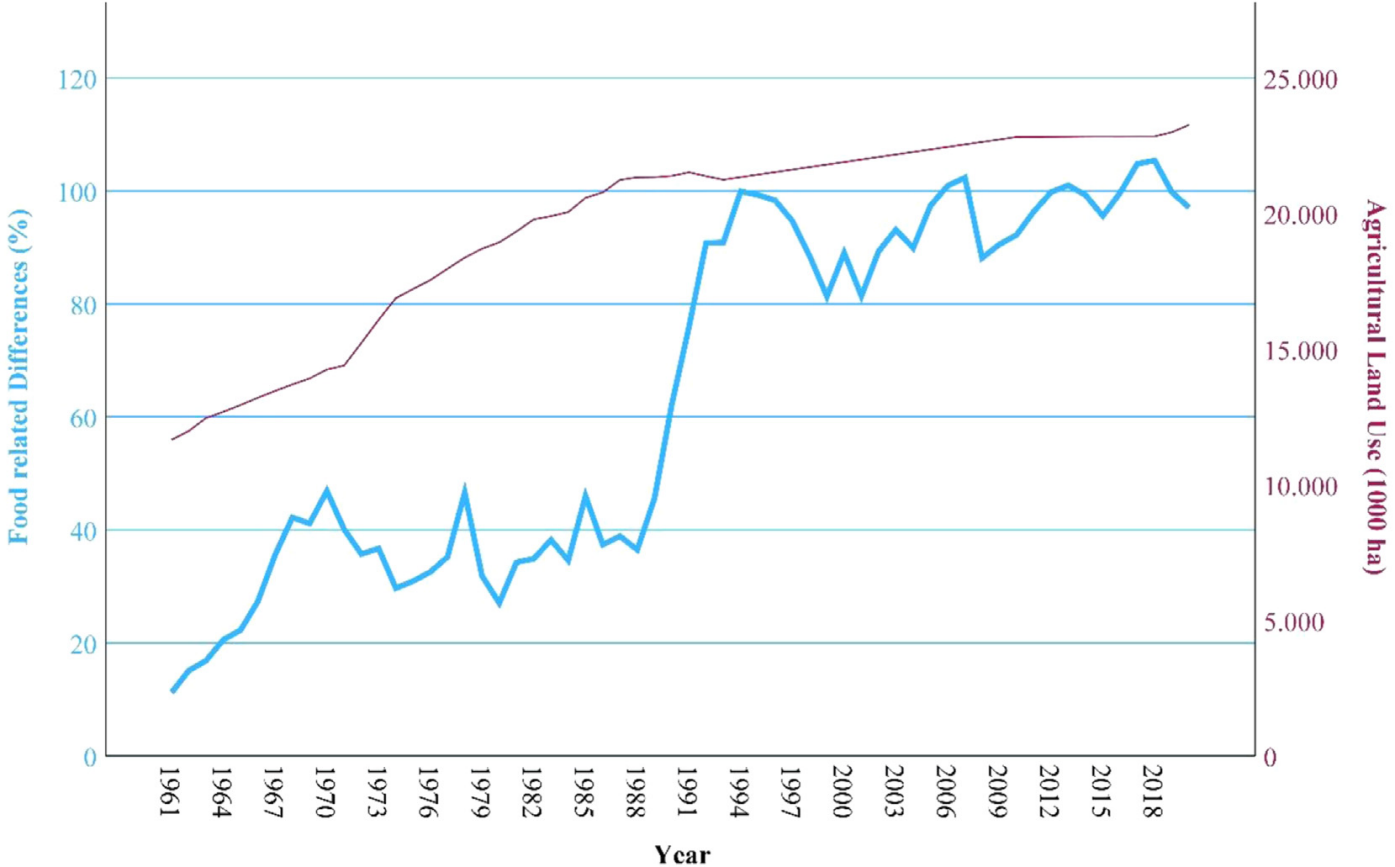
**Development agricultural land use and food related differences**. (Source: own source, based on EAT-Lancet-Commissions and FAOs data).

**Fig. 6:**
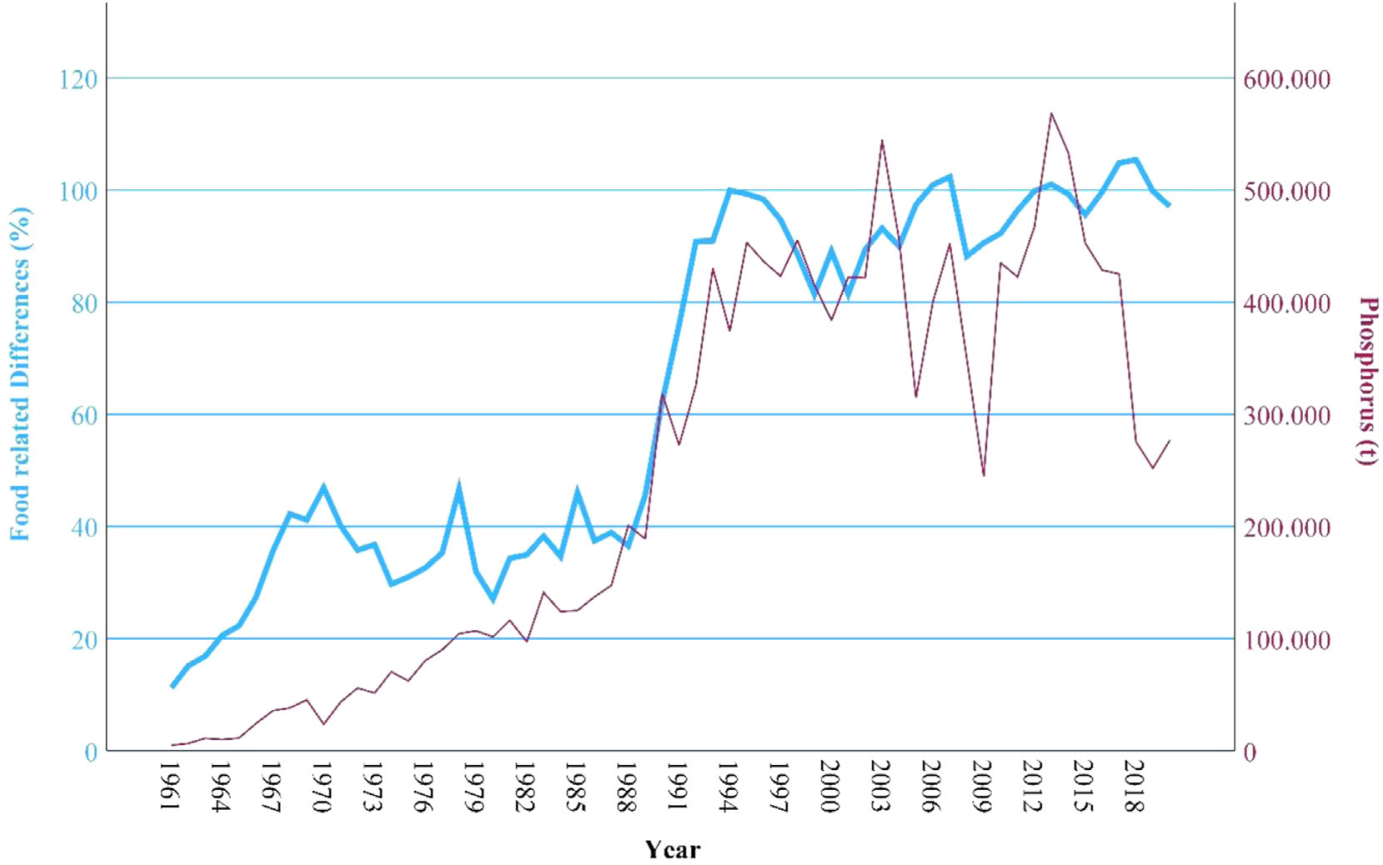
**Development phosphorus and food relateddifferences**. (Source: own source, based on EAT-Lancet-Commissions and FAOs data).

**Fig. 7:**
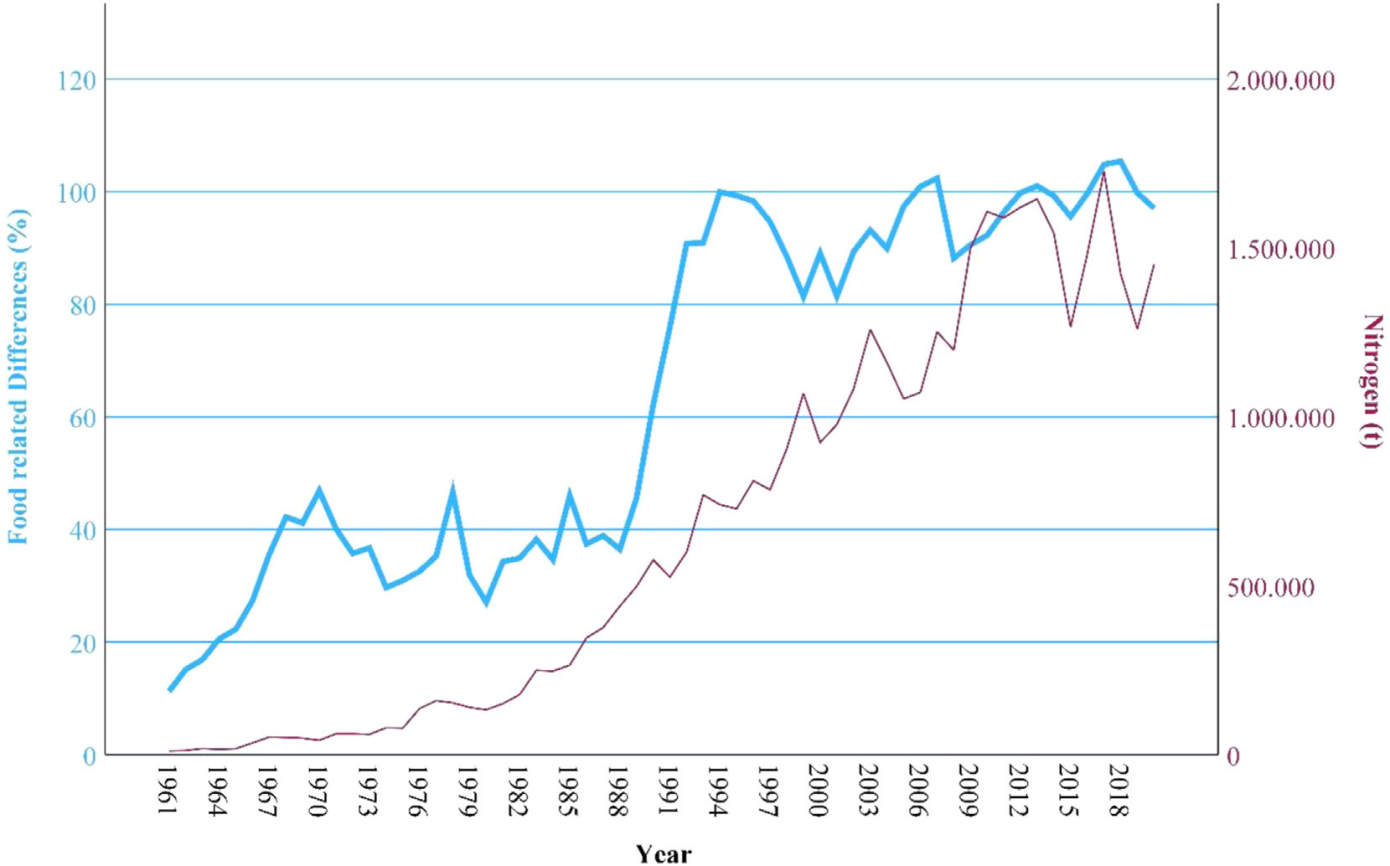
**Development Nitrogen andfood relateddifferences**. (Source: own source, based on EAT-Lancet-Commissions and FAOs data).

To identify potential correlations between altered dietary pattern and the incidence of NCDs as well as environmental impacts in Thailand, this study conducts a bivariate correlation analysis (Spearman correlation). The investigation is divided into two parts, including health impacts on Thai population (NCDs) and environmental impacts (GHG emissions, land use changes, nitrogen, and phosphorus inputs) in Thailand's agriculture. Two variables are compared for each, taking the changes between 1961 or 1990 and 2020 into account. The bivariate correlation analysis was conducted by the statistical software SPSS (version 18.0; SPSS, Chicago, IL, USA), considering the significance (p) and the correlation factor (r). For each correlation, the corresponding 95% confidence intervals (CI) are calculated. The width of the CIs reflects the degree of uncertainty around the correlation estimates, with wider intervals indicating greater uncertainty, particularly when smaller sample sizes are involved (in this case, the number of years observed). Since the development of NCD cases and GHG emissions was only observed over 30 years, the resulting CIs are wider, reflecting increased uncertainty in these estimates (see Table 2). Furthermore, linear charts are generated, which are showing the development of the respective dietary changes and NCD cases as well as environmental impacts over the last decades, visualizing correlations between the different variables (see Fig. 3, Fig. 4, Fig. 5, Fig. 6, Fig. 7).

#### Correlation between diet and health impacts

Concerning the relationship between dietary changes and health impacts in Thailand, two variables were compared between 1990 and 2020. Variable one describes the average of the previously calculated percentage differences from four food groups, which are identified as less healthy (independent variable). They include red and white meat, saturated oils as well as sugars.^1^ These food groups are known for promoting the development of NCDs, based on scientific evidence.^22^^,^^45^^,^^46^ The average of the previously calculated differences between these four food groups and PHD's mid-points, was calculated for each year between 1990 and 2020. Variable two describes Thailand's DALYs due to NCDs (dependent variable). The most significant risk factors for NCDs are unhealthy diets, physical inactivity, as well as tobacco and alcohol misuse. Since the occurrence of NCDs is strongly influenced by dietary habits, this indicator is used to illustrate the diet-related health status in Thailand.^47^ Results from the GBD were used for this variable as DALYs, which are presented as percentages for all NCD cases in Thailand's population.^43^ They take into account both, premature mortality and years lived with diseases or disabilities in reduced health. Various factors such as disease burden, quality of life, and mortality are combined into this single indicator, enabling a comprehensive assessment of health impacts. The NCDs include neoplasms, cardiovascular diseases, chronic respiratory diseases, digestive diseases, and musculoskeletal disorders, among others.^43^

#### Correlations between diet and environmental impacts

Concerning the relationship between environmental impacts and dietary changes in Thailand, the previously calculated differences from all animal-based food groups were used (independent variable), along with FAO's data, including GHG emissions, agricultural land areas, nitrogen and phosphorus inputs (dependent variables).^2^, ^3^, ^4^ In regard to environmental impacts, a total of four correlations, were calculated, each between the nutrition-related independent variable and the four environment-related dependent variables. The independent variable includes the average of the previously calculated differences of dairy, red and white meat, eggs and fish. Animal-based foods, are known to have more significant environmental impacts based on studies, while plant-based food groups are not considered due to their lower environmental impacts.^7^^,^^16^^,^^48^ The average of the percentage differences between these five food groups and PHD's mid-points, was calculated for each year between 1961 and 2020. The dependent variables describe four environmental impacts, which are important planetary boundaries that have already been exceeded. Other planetary boundaries were not considered in order to limit the paper's scope. Regarding GHG emissions, produced by Thailand's food production, CO_2_eq from CO_2_, CH_4_, and N_2_O are considered. These emissions are mainly generated by crop residues, rice cultivation, burning crops, synthetic fertilizers, pesticide manufacturing, on-farm energy use as well as food processing, packaging and transport.^3^ FAO provides full GHG emissions data only between 1990 and 2020, making the incomplete data before 1990 unusable.^3^ The GHG emissions data were summed together for each year from 1990 onwards. Data regarding land use changes, due to Thailand's food production, include agricultural land.^4^ Concerning land use changes in aquaculture and fisheries, there are no comprehensively data provided by FAO. Therefore, this dependent variable only consists of the available data on land use in food production in 1000 ha between 1961 and 2020.^4^ Concerning nitrogen and phosphorus use in Thailand's agriculture, FAO's data concerning fertilizers of nitrogen and phosphate were used, which are available between 1961 and 2020 with the unit of tons.^2^ The respective data were aggregated into each of the four dependent variables. Subsequently, four correlations between the independent and dependent variables were calculated using SPSS.

## Results

### Dietary and environmental changes

Grains in form of rice as well as fish and seafood are identified as the most important ingredients in Thailand's traditional cuisine. In addition, fruits, eggs, nuts (especially coconuts), and sugar are playing an important role and are consumed in large quantities until today.^17^^,^^18^^,^^35^ Thereby, the consumption quantities of meat, eggs, saturated oils, and sugar in particular increased significantly since 1961 (see Table 1).^17^^,^^18^ The food groups that have exceeded PHD's upper reference values include grains, red meat, eggs, saturated oils, and sugar, while vegetables and unsaturated oils fell below PHD's lower limits (see red-marked data in Table 1).

This development goes along with higher numbers of fast-food restaurants and conventional supermarkets, leading to larger consumption quantities of highly processed foods in Thailand.^32^ Highly processed foods, which are consumed in higher amounts today, include white polished rice, highly refined flour products (particularly in form of snacks, western style bakery items, and instant foods), fried potatoes, processed meats, pasteurized or sterilized coconut milk, fried or baked nuts, saturated oils as well as refined white sugar, and sweeteners.^27^, ^28^, ^29^, ^30^, ^31^^,^^49^ On the other hand, less processed food items are part of the traditional Thai cuisine, such as whole grain, hand-milled rice, natural or sun-dried fruits, health promoting herbs and spices (e.g. chilies, ginger, Thai basil, lemongrass, kaffir lime leaf or rind, galangal, coriander), handmade coconut milk as well as brown coconut syrup or sugar.^35^^,^^50^^,^^51^ These food items are associated with higher fiber- and nutrient contents, compared to highly processed foods, as well as health promoting properties.^35^^,^^51^^,^^52^

Beside of negative effects on human health due to changing dietary habits and food production practices, environmental impacts are on the rise.^53^ Due to agricultural intensification, more and more land is being converted into cropland (especially monocultures), leading to the loss of natural areas, such as forests as well as biodiversity and fertile soils.^37^ Furthermore, land use changes have contributed to increased GHG-emissions and pesticide use, presenting a potential risk to humans and the environment.^54^^,^^55^ From 1990 to 2020, GHG-Emissions (CO_2_eq) in Thailand's food production, increased from 124,627 to 190,538 kt.^3^ Especially animal products like meat, seafood, and dairy have negative effects on the environment due to increased land use, freshwater use, greenhouse gas emissions as well as nitrogen and phosphorus application, compared to plant-based foods.^7^^,^^16^ FAO's data show, that the use of land as well as nitrogen and phosphorus increased over the last decades. While in 1961 around 11,653 thousand ha were occupied by agriculture, it was already 23,272 thousand ha in 2020.^4^ In the same timeframe, the use of nitrogen fertilizer increased from 11,058 t to 1,451,653 t, while the amount of phosphate fertilizer increased from 4720 t to 276,801 t.^2^

A reason for these changes is intensifications of agriculture and animal farming due to rising demands of animal-based foods.^17^^,^^18^ Today, intensive beef production systems in Thailand are on the rise, which are using concentrate feed and houses.^56^ Compared to extensive systems, which are based on grazing and forage from grassland, intensive systems are consuming higher amounts of energy and lead to eutrophication as well as acidification to a greater extent.^56^ Rice, seafood, and fish are the main food sources in Thailand traditionally until today.^35^ However, the production of these foods has changed, due to modernized agriculture and manufacturing methods, causing rising environmental effects. 13.28 million ha or around 55.6% of Thailand's total agricultural area, are used for the production of rice.^57^ Especially rice fields are causing negative environmental effects, due to their emissions of CO_2_, CH_4,_ and N_2_O. The most N_2_O emissions are emitted through nitrogen fertilizer, while CH_4_ occur especially due to the fields flooding.^57^ Concerning CO_2_ emissions, the primary sources are burnings of crop residues and the use of machines for stationary or cropping operations, such as water pumping, applications of pesticides, land preparation as well as harvesting and tillage.^57^ Beside of rice farming, aquaculture production plays an important role in Thailand's agriculture and food culture.^35^^,^^58^ Almost 20 thousand ha of land is used for aquaculture farming, of which the highest yields are attributed to farmed shrimps with 40%, followed by fish (38%), and mollusks (22%).^58^ Due to unsustainable farming methods, this industry has caused environmental degradation such as water pollution, mangrove degradation, and biodiversity loss as well as lower yields.^58^ In addition to Thailand's own intensified agriculture and food production, larger quantities are imported from abroad to meet the growing demand for foods.^17^^,^^18^

### Percentage differences

Concerning the calculated percentage differences, the results in the positive range show five food groups, which are consumed in quantities, exceeding PHD's reference values. The largest positive differences in 2020, which are exceeding PHD's upper limit, occur in sugar (+452%), followed by red meat (+220%), and grains (+143%). Eggs (+19%), and saturated oils (+20%) show smaller differences in 2020 (see Fig. 1). All of these food groups developed towards the positive range or increased in it between the estimated time.

The results in the negative range show two food groups, which fall below PHD's lower limits in 2020. They include vegetables (−63%) and unsaturated oils (−61%). The food groups dairy (−84%), legumes (−82%), nuts (−47%) as well as tubers and starchy vegetables (−41%) also show strong negative differences from PHD's mid points. Nevertheless, they do not fall below the respective lower limits, since these are set at 0 g. Fruits show only a small difference with −12%. In case of tubers and starchy vegetables, dairy, legumes, and unsaturated oils, the differences decreased compared to 1961, while fruits and vegetables increased only slightly. The differences of nuts changed from +67% to −47% (see Fig. 2).

### Correlations

Concerning the bivariate correlation analysis, it was found that all investigated variables show significant correlations (see Table 2). This implies that the determined percentage differences of unhealthy or unsustainable food groups have increased concurrently with NCD cases and environmental impacts (GHG emissions, agricultural land-, nitrogen- and phosphorus use) over the last decades in Thailand.

**Table 2:** Correlations (r) and Significance (p) between all Variables.

	Correlation (r)	Significance (p)
NCDs	0.903 (95% CI 0.804-0.953)	<0.001
GHG Emissions	0.479 (95% CI 0.15–0.712)	<0.006
Land use	0.870 (95% CI 0.791–0.921)	<0.001
Phosphorus use	0.832 (95% CI 0.733–0.897)	<0.001
Nitrogen use	0.872 (95% CI 0.794–0.922)	<0.001

In regard to the two variables “food related differences” and “NCDs based on DALYs”, there is a significant, high positive correlation observed through the bivariate correlation analysis. Over the 30 estimated years, the Spearman correlation (r) between the two variables, accounts to r = 0.903 (95% CI 0.804–0.953), while the significance (p) is p ≤ 0.001 (Table 2). The evolution of the food related differences and NCDs in DALYs can be observed in Fig. 3.

Between the variables “food related differences” and “agricultural impacts (GHG emissions; land use, phosphorus use; nitrogen use)” there are significant (p), high or very high positive correlations (r) detected through the bivariate correlation analysis (see Table 2). The development of the food related differences in comparison with Thailand's agricultural impacts can be observed in Fig. 4, Fig. 5, Fig. 6, Fig. 7.

## Discussion

The results show, that the most significant differences between the PHD and Thailand's average dietary pattern, are the increased consumption of red meat, grains, saturated oils, eggs, and particularly important, sugar as well as the low consumption quantities of vegetables and unsaturated oils. Chicken and other poultry, fish, seafood, and dairy products did not exceed the upper limits of the PHD, which is a positive aspect in terms of environmental and human health.^7^ However, production techniques, particularly in seafood and fish production, are becoming increasingly unsustainable.^58^ Implementing sustainable practices in agriculture and food production would be advantageous. Examples in this case are marine protected areas, selective fishing techniques, and sustainable aquaculture practices.^58^, ^59^, ^60^ Further sustainable actions in food production are the implementing of sustainable phosphorus and nitrogen use, renewable energy, and the reduction of chemical pollution due to herbicides and pesticides.^7^ While fruits are consumed in sufficient quantities, an increase in legume-consumption might be beneficial, due to their low environmental impacts and health promoting qualities.^7^^,^^61^ Since the consumption of nuts has declined over the past decades, an increased intake could also be beneficial.^7^^,^^62^ It is worth noting that, compared to other countries, there is less consumption of white and red meat and an adequate intake of fruits.^17^^,^^18^^,^^40^ However, a reduction in red and processed meat would benefit human and planetary health. Since rice has traditionally been one of the most important food sources in Thailand until today, grains are exceeding PHD's reference values.^35^ However, it should be noted, that smaller amounts of tubers and starchy vegetables are consumed, which might make it reasonable to set the consumption recommendations for whole grains in Thailand higher than those of the PHD.^7^^,^^17^^,^^18^ Nevertheless, the intake, particularly of white rice and highly refined flour, should be reduced.

Thailand's dietary guidelines, are not current and focus on promoting human health, while sustainable aspects are not considered.^63^ Therefore, research and results such as those presented, could be considered in the formulation of current dietary guidelines to support sustainable development in Thailand and Asia. These may include a reduction in sugar, red meat, grains, saturated oils and eggs, along with an increase in vegetables, and unsaturated oils. Due to the excessively high consumption of sugar, special attention should be paid to this aspect. The present literature review found that the traditional Thai cuisine incorporates less processed and more natural foods, along with health promoting herbs and spices as well as a more sustainable food production.^34^, ^35^, ^36^^,^^50^, ^51^, ^52^, ^53^ Therefore, an approach that combines traditional dietary habits with modern and sustainable agricultural and production techniques may be advantageous to support human and environmental health. Policy makers, in the fields of public health and agriculture, can benefit from the results, especially in Thailand, South East Asia or countries with rising economic growth. Consequently, the findings can contribute to the development of new agricultural and public health strategies, as well as current dietary guidelines.

While the percentage differences of less healthy and sustainable food groups increased over the last decades, NCD cases as well as environmental impacts (GHG emissions, agricultural land-, nitrogen- and phosphorus use) have risen concurrently.^2^, ^3^, ^4^^,^^7^^,^^17^^,^^18^ Since food production and dietary habits are given a significant impact on human and environmental health, a connection may exist.^7^ However, the present study only investigates correlations, precluding the derivation of causality.

Limitations of this study, mainly refers to FAO's data, which are presented in “supplies.” These quantities are not equivalent to actual consumption quantities, which limits the interpretability of the results. The supply data, used in the present work, reflects the total amount of food, which is available for consumption in Thailand. They do not account for trade, food waste or individual dietary choices. Furthermore, the considered data do not provide insights into the preparation and processing of food items, which makes it more challenging to assess the health and environmental impact. Additionally, various data on GHG emissions are unavailable in FAO's dataset. As a result, only GHG emissions data from 1990 onwards were used in this study, which limits the sample size and contributes to the wider CI observed. This may weaken the significance of the correlation, as the broader interval indicates greater uncertainty in the estimate. FAO's data collection does not include direct data on biodiversity loss either. To ensure a consistent data source for environmental impacts, the assessment of biodiversity loss was therefore excluded in this study. Nevertheless, this planetary boundary is an important factor, since further exceeding might result in significant environmental degradation. Future research could examine how Thailand's dietary pattern relate to biodiversity loss, taking changes over time under account. Moreover, the FAO does not provide data on land use changes in aquaculture and fisheries. As a result, these factors were excluded from the analysis. Considering that aquaculture and fisheries are important factors in Thailand's agriculture, the actual agricultural land use may be substantially higher. Concerning the GBD's data, there are uncertainties and controversies due to substantial use of imputations and modeling, driven by data gaps in certain regions and for various causes, as well as the assignment of disability weights based on subjective evaluations.^64^ Furthermore, the data only extend back to 1990, which may weaken the significance of the observed correlation. In regard to the correlations of GHG emissions, land use as well as nitrogen and phosphorus, the growing world population is not considered, what might lead to higher correlations in this study.

Since the whole population and environment of Thailand was considered, no subgroup analyses or multivariate models have been conducted in this study, which could account for factors such as age, gender, education, and income of the Thai population. However, these models would be beneficial to go beyond the calculated correlations and gain a deeper understanding of the complex relationship between food consumption, NCDs, and environmental aspects. This approach would allow for the consideration of different confounders, improving the accuracy and consistency of the results. Further research could also apply data, which consider the origin and preparation of food groups and do not rely on supply data, for example through consumption surveys.

However, the results and corresponding dietary recommendations could be beneficial for Thailand and other countries, particularly those, who are classified as upper- and middle-income countries with rising economic growth. In summary, this study has achieved significant results that can support sustainable development.

## Contributors

AB contributed to the conceptualization, data curation, formal analysis, investigation, methodology, resources, visualization, and wrote the original draft. She also participated in the review and editing process. CS provided supervision and contributed to the conceptualization as well as the review and editing of the manuscript. KK was involved in the data curation, supervision, resources, and the review and editing of the manuscript. All authors had access to the raw underlying data. KK made the final decision regarding the submission of the manuscript.

## Data sharing statement

The data used in this study includes publicly available datasets from the FAO and GBD study related to food supply, NCD cases, and environmental impacts (greenhouse gas emissions, land use, nitrogen, and phosphorus). These datasets cover the population or agriculture of Thailand and are openly accessible. Personal identifiers are not included. The available data can be freely used by other researchers. However, proper citation of the original sources is required for any further use or analysis.

## Declaration of interests

The authors declare that they have no competing interests.

## Appendix A Supplementary data

Thai Abstract
