# Supplementary material for: Thailand – how far are we from achieving a healthy and sustainable diet? A longitudinal ecological study
Source: Lancet Reg Health Southeast Asia. 2024 Sep 13;29:100478. doi: 10.1016/j.lansea.2024.100478 (PMC11418144; doi:10.1016/j.lansea.2024.100478)
Supplement: Thai Abstract [file mmc1.docx]

*This translation in [language] was submitted by the authors and we reproduce it as supplied. It has not been peer reviewed. Our editorial processes have only been applied to the original abstract in English, which should serve as reference for this manuscript.*

**บทคัดย่อ**

ความเป็นมา: ประเทศอุตสาหกรรมใหม่ เช่น ประเทศไทย ได้รับอิทธิพลจากโลกาภิวัตน์ ความเป็นตะวันตก และการขยายตัวของเมืองในช่วงหลายทศวรรษที่ผ่านมาส่งผลให้เกิดการเปลี่ยนแปลงในพฤติกรรมการกินและการผลิตอาหาร ผลที่ตามมาของการเปลี่ยนแปลงเหล่านี้ ได้แก่ โรคเรื้อรังไม่ติดเชื้อ (NCDs) ที่เพิ่มขึ้นและความเสื่อมโทรมของสิ่งแวดล้อม ซึ่งถือเป็นความท้าทายระดับโลกที่สำคัญที่สุดในปัจจุบัน วัตถุประสงค์ของการศึกษานี้คือเพื่อวินิจฉัยการเปลี่ยนแปลงด้านโภชนาการของประเทศไทย โดยพิจารณาจากด้านสุขภาพและด้านความยั่งยืน อีกทั้งเพื่อกำหนดความสัมพันธ์ระหว่างการเปลี่ยนแปลงเหล่านี้กับการเกิดโรคเรื้อรังไม่ติดเชื้อ รวมถึงผลกระทบต่อสิ่งแวดล้อม (การปล่อยก๊าซเรือนกระจก การใช้ที่ดิน ไนโตรเจน ฟอสฟอรัส) โดยวิธีนี้ จึงสามารถวินิจฉัยการปรับเปลี่ยนที่เกี่ยวข้องกับอาหารเพื่ออนุรักษ์โลกและส่งเสริมสุขภาพมนุษย์ได้

ระเบียบวิธีวิจัย: ในการศึกษาทางนิเวศวิทยานี้ ได้ทำการคำนวณความแตกต่างสัมพันธ์ระหว่างการบริโภคอาหารโดยเฉลี่ยและค่าอ้างอิงของอาหาร Planetary Health Diet (PHD) นอกจากนี้ ยังมีการทำการวิเคราะห์สหสัมพันธ์ โดยใช้ข้อมูลจากองค์การอาหารและ

เกษตรแห่งสหประชาชาติ (FAO) ผลการศึกษาภาระทางสุขภาพโลก 2019 (GBD) และ

ค่าอ้างอิงของ PHD

ผลการศึกษา: ปริมาณการบริโภคเนื้อสัตว์ ไข่ น้ำมันอิ่มตัว และน้ำตาลเพิ่มขึ้น

อย่างมีนัยสำคัญตั้งแต่ปี 1961 กลุ่มอาหารที่เกินค่าอ้างอิงสูงสุดของ PHD ได้แก่

ธัญพืช (+143%) เนื้อแดง (+220%) ไข่ (+19%) น้ำมันอิ่มตัว (+20%) และน้ำตาล

(+994%) ในขณะที่ผัก (-63%) และน้ำมันไม่อิ่มตัว (-61%) ต่ำกว่าขีดจำกัดล่าง

ของ PHD สำหรับการวิเคราะห์ความสัมพันธ์แบบสองตัวแปร ตัวแปรที่ตรวจสอบ

ทั้งหมดแสดงให้เห็นถึงความสัมพันธ์ที่สำคัญ พบความสัมพันธ์ที่สำคัญที่สุดในการ

ใช้ไนโตรเจน (r=0.872, 95% CI 0.794-0.922) และการใช้ที่ดิน (r=0.870, 95%

CI 0.791-0.921) รองลงมาคือการใช้ฟอสฟอรัส (r=0.832, 95% CI

0.733-0.897) การเกิดโรค NCD (r=0.782, 95% CI 0.587-0.891) และการปล่อยก๊าซ

เรือนกระจก (r=0.479, 95% CI 0.15-0.712)

การอธิบาย: ผลการศึกษาแสดงให้เห็นว่าความแตกต่างที่กำหนดของกลุ่มอาหารที่

ไม่ดีต่อสุขภาพหรือไม่ยั่งยืนเพิ่มขึ้นพร้อมกันกับการเกิดโรค NCD และผลกระทบต่อสิ่ง

แวดล้อมในช่วงหลายทศวรรษที่ผ่านมาในประเทศไทย การเปลี่ยนแปลงไปสู่การ

ลดการบริโภคธัญพืช เนื้อแดง ไข่ น้ำตาล และไขมันอิ่มตัว รวมถึงการเพิ่มปริมาณ

ผักและน้ำมันไม่อิ่มตัว อาจช่วยอนุรักษ์สิ่งแวดล้อมและส่งเสริมสุขภาพของมนุษย์ได้
